# Notes on Medical Matters and Medical Men in Paris

**Published:** 1847-05

**Authors:** David W. Yandell

**Affiliations:** Louisville, Ky.; Paris


### WESTERN JOURNAL

OF

### MEDICINE AND SURGERY.

MAY, 1847.

**Art. I.**—*Notes on Medical Matters and Medical Men in Paris.* By David W. Yandell, M.D., of Louisville, Ky.

I had the pleasure in a former letter of giving you an extract from the memoir of Dr. Nélaton, on the very interesting subject of *Cancer of the Bones;* the whole of the memoir has since appeared in the Annual of Surgery and Medicine, and is so new and valuable that I have translated it for the Journal, a task the more readily undertaken for knowing the very high estimation in which the author is held by his brethren. I have only to regret that he cannot be allowed to speak to your readers in his own language, in which he would appear, I doubt not, to far more advantage than I shall be able to exhibit him in an English garb.

*Memoir on Cancer of the Bones.* By Dr. Nélaton, Professor to the Faculty of Medicine of Paris.—We find cancer of the bones, generally designated *osteo-sarcoma*, described, by the authors of the last century, as J. L. Petit, Duverney, and Heister, who continually confound it with caries and exostosis, under the name of *carnification des os.* Among modern writers, Sir A Cooper, under the title of *fungous exostosis* of the medullary membrane, described one of the varieties of cancerous degeneration of the bones, and although Boyer has written of laminated cellular exostosis, it does not prevent him from afterwards devoting a chapter to osteo-sarcoma, affections between which one in vain endeavvors to detect differences which in reality do not exist. In a third article, where he treats of spina ventosa of adults, the same writer describes one of the forms of cancer of the bones. It is easy to perceive that different names given to the same disease must create doubt and uncertainty in the mind of the reader. Cancer, it is true, does not always produce the same disorders in the bones, or to speak more accurately, does not always manifest itself by the same symptoms; nevertheless we are bound to acknowledge that in spite of these differences, the essential characters of the disease do not vary, and that the stamp of cancerous affections is constantly present. The different forms which it offers should be united under the same general title; this work will thus comprehend the history of cellular exostosis, spina ventosa of adults, and osteo-sarcoma.

Cancer is developed sometimes primitively in the osseous tissues; at other times again this tissue is attacked consecutively, in consequence of the propagation of a degeneration which originally commenced in the neighboring soft parts.

*Anatomy and Physiological Pathology.*—We have already remarked that cancer of the bones presents itself under different forms, which may be reduced to four:

*First form.* We find in the interior of the osseous tissue the nuclei of the cancerous growth forming round patches contained in excavations which they exactly fill; the tissue of the bone has completely disappeared in the points invaded by the accidental substance, and is replaced by the cancerous matter; there is a loss of osseous substance similar to that produced by a cutting punch; this destruction observed equally in the compact and spongy portion is sometimes seen on the bones of the cranium, on the sternum and ribs, and on the diaphyses of the femur and humerus. In the neighborhood of this loss of substance the bone appears to have suffered no alteration, and but little more vascularity is produced than is consistent with the healthy state. In the beginning the cancerous nucleus preserves exactly the form of the portion of bone which has disappeared; and it seems that the progress of the degeneration is limited by the periosteum; at a more advanced period, however, the cancerous matter increases in development, and forms a more or less considerable prominence on the surface of the altered bone. But if the seat of the accidental production be a long bone and correspond to its diaphysis, as the medullary membrane does not offer the same obstacles to its growth as the periosteum, it is not uncommon to see the cancerous mass extend towards the medullary canal in which it propagates itself with rapidity, and thus forms in its interior a plug or stopper, which extends much higher than the tumor which projects from the surface of the bone would lead one to suppose — a disposition, I need hardly remark, which it is extremely important to bear in mind when it is decided to amputate the limb in this variety of the affection.

*Second form.* In this division the tissue of the bone has undergone a profound alteration and presents a tumor, generally voluminous, a section of which exposes a great number of cells, very irregular in form and dimensions, filled by cancerous matter in various stages of softening; there seems to be a considerable diminution of the osseous tissue in the cells of which the cancerous substance is deposited. This variety is met with particularly on the epiphysis of the long bones; the tumor increases not unfrequently very slowly, and often acquires considerable size. This is the form in which tumors weighing twenty, thirty, and even fifty pounds have been found, and is the affection which has been more especially described in standard treatises as *osteo-sarcoma.*

*Third form.* In this the cancerous mass, commencing in the interior of the bone, gradually increases, but instead of destroying the osseous tissue, as is the case in the first form, instead of producing a cellular structure, as in the second, it presses it eccentrically, causing it to yield in a slow and gradual manner, and ends by presenting a very thin and friable shell, in the interior of which the cancerous matter is contained. This is the *spina ventosa* of authors. This species of cancer, more rare than the preceding, is confined almost exclusively to the long bones of the extremities, although it has been occasionally observed on the inferior and superior maxillary bones. When the tumor has acquired sufficient volume, the osseous shell becomes perforated, the tumor escapes by the perforation, approaches the surface of the body, and ultimately progresses like the other varieties.

*Fourth form.* The tumor is applied to the exterior of the bone and covered by the periosteum, but it is very easy to perceive that the osseous tissue is itself altered, in fact, a collection of excessively fine, flexible osseous prolongations similar to hair implanted on the surface of the bone are observed, which are often three or four centimeters in length, placed one against the other so as to represent a bundle, not unlike the wick of a candle, in the intervals and on the surface of which the cancerous matter is deposited. This disposition, more rare than the foregoing, has been frequently observed upon the iliac bones. At the moment I am writing this, there is before me a femur which affords a very striking example of this variety. This tumor, which possessed considerable size before maceration, envelops the whole inferior third of the bone, and is covered with osseous points three or four centimeters (about an inch) in length.

Such are the different forms assumed by cancer of the bones. Well marked differences, it will be perceived, exist between them, while at the same time no one is deficient in the characters proper to cancerous affections. Thus, whatever may be its first form, the tumor constantly increases, softens, ulcerates, is reproduced almost always after removal, and terminates by inducing the cancerous cachexia.

An important remark to be made in relation to the development of these tumors is, that they never affect the cartilaginous tissues; for example, when the cancer commences near the articular extremity of the bone, the diarthrodial cartilage is found untouched, notwithstanding the complete degeneration of the epiphysis which supported it. This immunity of the diarthrodial cartilage renders the propagation of the cancer into the articular cavity of very rare occurrence; still it does sometimes happen, and the following is the process by which it is effected: The cancerous matter being arrested in its course by the cartilaginous lamina increases laterally, until it arrives at a point where the osseous tissue is covered only by periosteum and synovial membrane; these two tissues are soon attacked by cancer; the tumor advances into the interior of the articular capsule, which it distends in a manner similar in certain cases to that observed in effusion of liquids. I have shown this year to the Society of Surgery an extremely fine example of this propagation of a cancer of the femur into the articulation of the knee. In this case the accidental production was introduced into the articulation by the space which separates the two condyles of the femur.

Until now I have adhered to the somewhat vague designation of cancerous tissue, reserving for myself an explanation upon this point; in an immense majority of cases it is the *encephaloid* tissue in various degrees of development, that is observed in the bones. Sometimes it shows itself under the form of quite fine white or rose colored and slightly vascular masses, at others forming an extremely soft, almost diffluent tumor, traversed by a great number of vessels, presenting occasionally in its interior sanguine collections and fibrinous clots more or less altered.

In a few rarer cases it is the *colloid* tissue which constitutes the morbid mass; still it is more frequently allied to the encephaloid tissue. It is not uncommon, particularly in the second form of the disease described, to see in the same tumor these two tissues in different stages of development, besides which, true cysts containing a serous or sero-sanguinolent fluid are often found.

Finally, melanosis has been encountered in the bones, but always, so far at least as I am informed, in a state of infiltration. Strictly speaking, this would constitute a fifth variety of cancer of the bones; but it has never attracted the attention of surgeons, since it in no way modifies the texture of the bone, and does not manifest itself by any characteristic symptom. It is proper to remark that the scirrhous tissue which is frequently found in other parts of the body never shows itself in the bones, at least I am not aware that the presence of this accidental tissue in these parts has ever been clearly established by observation. In certain cases the encephaloid tissue has been found, according to some authors, mixed, so to speak, with an erectile tissue, which would offer a combination of two morbid tissues.

*Symptomatology.* Although the four different forms of cancer now described do not present a series of symptoms precisely similar, we believe it preferable to exhibit collectively the symptomatology of the various forms, seeing that they possess a great number of points in common, and that a special description of each would lead us into unnecessary repetitions, adding that the reader, in recalling what we have said in the preceding paragraph upon the anatomy and the physiological pathology, must easily recognise in the exposition the characters which belong to each variety.

Some patients experience at the beginning of the affection, for a longer or shorter time, pretty vivid but passing pains, which recur sometimes spontaneously, at other times supervene after fatigue or sudden movements; still nothing is revealed on an examination of the painful part; but after a certain time a tumor begins to show itself. In other patients, on the contrary, the tumor appears without having been preceded by pains; it is indolent and scarcely attracts the attention of the patient, but in either case it increases, sometimes slowly, sometimes with frightful rapidity.

When the tumor is of small volume, the skin which covers it preserves its normal aspect, and from its transparency only allows to be seen certain flexuous and dilated veins; but if the cancerous mass has attained great size, the skin is distended, glossy, bright and attenuated; it is then especially that the subcutaneous veins assume the form of sinuous, fine lines, which give the finger on exploration the sensation of grooves more or less deeply excavated on the surface of the tumor. This presents, in certain instances, a softness which suggests the idea of a collection of liquid, while at other times it is hard, and offers an osseous resistance. Its surface, at first quite regularly round, loses its form; prominent and soft nipples are here and there remarked, while others are more firm and resisting under the finger. Between these various portions are occasionally observed harder points, formed by the osseous enclosure or partitions which divide the tumor into numerous segments.

In the second form of cancer of the bone described, in which the tumor occupies the whole circumference of the bone, an exceedingly curious phenomenon, and one which possesses a certain importance in the diagnosis, is observed: The tumor, formed by a great number of osseous cells, increases in a lateral direction especially, but at the same time is developed in a line parallel with the axis of the bone; the result of which is that the two extremities gradually separate, and the whole of the bone undergoes a true lengthening, which in some instances has been as great as five or six centimetres.

If endeavors are made to move the tumor, it is perceived that the motions are transmitted to the subjacent bone, which seems to constitute a part of the tumor. In some cases a deformity and swelling of the bone at the point where it unites to the base of the tumor, may be recognised by the touch. In certain other cases a peculiar sensation is imparted to the touch, which has been compared to the rumpling of dry parchment or the crushing of an eggshell; this sign belongs especially to the third form of which we have spoken, though it may also present itself in the second, when pressure has been exercised on one of the fragile partitions which we have described; and it may possibly be met with even in the first form, when an extremely delicate lamella still covers the cancerous nucleus developed in the interior of the bone. This sign can be by no means always perceived; and it is worthy of mention that after this crepitation has been elicited a certain number of times, it can no longer be produced until at the end of some twelve or twenty-four hours, when it may be detected anew.

Other tumors, quite rare, it is true, offer beats synchronous with the arterial pulsations—beats which do not consist in a simple rising, but in a true expansive movement of the parts, as is met with in aneurismal tumors. The ear applied to the diseased region perceives frequently in this case a *bruit de souffle* more or less marked, but still, ordinarily, not nearly as distinct as that of aneurismal tumors. The patients experience lancinating pains in the tumors, which are often more severe in the night than day, and might suggest the idea of a syphilitic cause, if it were not known that this is a character proper to the greater part of organic affections of the osseous tissue. Some patients nevertheless feel no pain, and are, so to speak, troubled only by the enormous volume of the limb; while it is a matter of surprise to how great an extent the subjects of these tumors retain the power of moving the joints contiguous to the diseased part. It is easy to understand how the bones, profoundly altered by the cancer, should break in certain cases with extreme facility; in fact, a sudden movement or even an inconsiderable strain is sometimes sufficient to fracture the most resisting bones—the femur, for example; and the prognosis should always be grave under such circumstances, for the fractures do not ordinarily unite.

Independently of the symptoms proper to the tumor—symptoms manifested, no matter in what part it may be situated—others are observed, which depend upon the compression of the neighboring organs. These symptoms of neighborhood, as they are called by M. Gerdy, vary according to the seat of the tumor; thus there will sometimes be an œdema dependent on compression of the principal venous trunks; at others, neuralgic pains, arising either from distension or compression of certain nervous filaments; in fine, the most varied functional troubles, such as dyspnœa, dysphagia, retention of urine, etc., may be manifested as the trachea, œsophagus or urethra may be displaced or compressed by the tumor developed in their neighborhood. In certain cases these symptoms alone suffice to jeopard life; but in cases where the tumor does not embarrass the functions of any organ indispensable to life, it continues to be developed, and presents the whole series of phenomena belonging to cancerous affections; thus ulceration, hemorrhages and the cancerous cachexia supervene, and finally death occurs. When the tumor is of any great volume, as is observed frequently in the second form described, it is not uncommon to see these phenomena occur and succeed each other with frightful rapidity, although the tumor may have increased up to this point with extreme slowness. The steps by which it is effected are these: The skin, which was violently distended, is attacked by erysipelas; one or more gangrenous patches appear on different points of the tumor; these become detached and lay bare the softened cancerous tissue; an ichorous liquid flows from the surface of the ulcerated parts; finally the patient succumbs, exhausted by the pains and losses which he every day experiences.

*Diagnosis.*—When there does not yet exist any tumor appreciable to the eye or touch, the pains which accompany the development of the cancer may be taken for those of rheumatism or syphilis; nor is it difficult to perceive how such a mistake might occur, since cancer of the bones is an affection rarely met with, while rheumatism and syphilitic diseases are extremely common. The latter will naturally first suggest themselves to the mind of the physician. This error may be avoided by considering that rheumatic pains are generally erratic, that they cease for several weeks and even months, to reappear and continue uninterruptedly for several days; while the pains proper to cancer show themselves constantly in the same point, and do not, like the preceding, reappear under the form of paroxysms. As to the pains in the bones incident to syphilis in certain forms, their exacerbation, produced either by the heat of the bed or pressure of the fingers, the anterior existence of syphilis, and the influence of an antivenereal treatment, will generally lead to their recognition.

When the tumor begins to show itself, it may be taken for a periostosis; but this is accompanied and has usually been preceded by characteristic pains, and, moreover, promptly yields to the proper treatment.

When the tumor has attained greater size, it may be confounded with either an exostosis, a cyst developed in the osseous tissue, an aneurism, or, finally, with those pulsating tumors described under the name of aneurism of the bones. But it is especially osteo-cartilaginous exostosis that is apt to be confounded with cancer. A considerable degree of hardness, the absence of pains, and an extremely slow march, render the existence of an exostosis very probable; whereas the circumstances the opposite of these would lead one to infer the presence of a cancer. But we must confess that the diagnosis will often present, in such a case as this, an uncertainty which can only be dissipated by a prolonged attention to the progress of the disease.

There is a strikiug resemblance between cysts developed in the osseous tissue and the third form of cancer described; in the two cases the laminæ of the osseous tissue are crowded together, thinned, and afford to the finger on pressure a crepitation; and an explorative puncture is the only means of resolving the doubt. If the records of science did not contain a great number of cases in which we see cancerous tumors have been mistaken for aneurisms, one could scarcely conceive how such errors could be committed. Nevertheless it ceases to be a matter of surprise, when it is remembered that certain cancers of the bones present pulsations synchronous with those of the arteries, and offer also a *bruit de souffle.* But it is necessary to remark that these tumors present these two phenomena only at a period when the accidental tissue has become exceedingly vascular—that is to say, at an advanced stage of its development—while the pulsations in aneurism are perceived from its beginning. It is here proper to recall that circumstance, quite rare, it is true, of which we have spoken in the pathological anatomy of this affection, viz., the lengthening of the bone witnessed in some examples of cancer, and which is never seen in any case of aneurism.

Every cancer of the bones is an extremely grave affection; but the danger varies according to its seat, its degree of development, and its progress, which, in the second and third forms, is much slower than in the remaining two. Often, in the latter case, there exists but a single tumor, well limited by an osseous envelop, which circumstance may facilitate the removal of the degenerated bone; whereas in the first, on the contrary, there generally exist multiplied nuclei, a condition which renders all treatment comparatively hopeless.

*Treatment.*—This is divided into curative and palliative. The first consists in complete removal of the tumor; to render which operation practicable, certain local conditions are indispensable: 1st, we must be able to determine with exactitude the extent of the tumor and its connections with the neighboring organs; 2d, that the soft parts are sound to an extent which allows the formation of flaps sufficient to cover the wound succeeding the removal of the degenerated bone.

When the tumor is situated on one of the limbs, amputation or disarticulation is to be resorted to—some writers esteeming the latter preferable, because it is not liable, as amputation is, to the objection that it may leave a cancerous prolongation in the medullary canal above the point of section. But this otherwise judicious observation is applicable only to the first form of the d[illegible]ase; and even in this variety it is not wise, in anticipation of a danger which after all may not exist, uselessly to sacrifice a considerable portion of a member, and thus render the operation much more serious. It is generally sufficient to amputate two or three inches above the tumor, immediately removing another portion of the bone if it is ascertained that the cancer has propagated itself in the medullary canal to the point of the first section.

At other times, when the bone affected is of small dimensions, it is extirpated entire; it is in such instances that the bones of the metacarpus, metatarsus, and even the clavicle have been repeatedly disarticulated with success. Finally, in certain cases, one may confine himself to an exsection of the altered portion of the bone, an operation which has been practised a great number of times with success in the inferior and superior maxillary bones.

If the situation or the volume of the tumor prevents a complete removal, a palliative treatment is alone applicable. The patient should avoid with the greatest care every cause likely to induce a rapid progress in his affection, such as the use of topical irritants with the view of obtaining resolution; he should abstain even from every local application, at least while there are no acute pains. When these affect the patient, simple cataplasms sprinkled with opium afford more relief than anything else. If this is not sufficient, unguents containing the extract of opium or belladonna, or the salts of morphine, applied by the endermic method, may sometimes be advantageously employed. The narcotic preparations ought to be given internally also; but it is proper to add, that these remedies soon lose their power to calm the pain, and that it is necessary to change them often or to increase the size of the dose.

The following article will be found to be one of unusual interest. It has been sought after by the Paris physicians with the greatest avidity, and is here esteemed one of singular ability. It is entitled—

*Clinical Researches concerning Phlebitis, especially that called Spontaneous Phlebitis.* By C. Forget, Professor of Clinical Medicine of the Faculty of Strasbourg.—Phlebitis is a disease that has been known only a short time, and which notwithstanding the numerous investigations to which it has already given rise still presents a great number of problems that are unresolved. It would even seem that science has retrograded on this subject, for various points in the history of phlebitis that not long ago were admitted to be substantiated, are again made matters of discussion. Thus, the formation of a sanguine clot as a necessary phenomenon of phlebitis is contested; as also, that this clot should be considered as the cause and not rather the effect of the disease; and a dispute has arisen as to the reality of the transmission of pus into the circulatory channels. My object is not to reply to these questions, but I shall confine myself simply to those upon which I believe I may throw some light, and which appear to me to have some importance in a practical point of view.

Phlebitis has been divided into suppurative and non-suppurative, adhesive and non-adhesive, traumatic and spontaneous, etc. Although these various denominations are said to be based on fundamental differences between the species, in our opinion they are but accidents of the same affection, and express nothing; still they possess a certain practical utility; and it is the last form, I think, which offers the greatest interest, if the power, that we believe may sometimes be attributed to the contact of the air, be admitted as real. We shall treat separately in this essay of traumatic and spontaneous phlebitis.

1. *Traumatic Phlebitis.*—Previous to the works of modern writers on inflammation of the veins, the inflammatory accidents following traumatic lesions, bloody operations, and particularly venesection, were attributed to anything else than to phlebitis and purulent infection; but since then every local inflammation supervening after bleeding has been considered as almost always due to a phlebitis, and every phlebitis has been regarded as almost always necessarily mortal. The first of these opinions is an error in diagnosis that the instructed practitioner knows how to avoid; the second is an exaggeration suggested by the terrors that purulent infection may well inspire, against which opinion, however, there is a large array of facts, to which we desire to add our modest share. The first of these questions scarcely meriting a serious examination, we will pass rapidly over it.

1st. *The inflammations which result from the operation of venesection are not always phlebitis.* And, in fact, among nine cases of grave inflammatory accidents observed by ourselves after phlebotomy, in three these accidents have consisted of simple phlegmons.

Case I.—In a man affected with albuminuria, a bleeding at the arm was followed by an intense phlegmon, of which the repeated application of leeches and topical emollients and sedatives procured the resolution in the space of a few days.

Case II.—Venesection practised in a woman laboring under pneumonia was followed by the development of a grave phlegmon at the bend of the arm, which, notwithstanding antiphlogistics were extensively applied, terminated by suppuration. Incisions suitably made gave issue to pus, and recovery took place without any general accidents, five weeks after the appearance of the phlegmon.

Case III.—Bleeding at the arm in a man 68 years old, suffering from bronchitis, gave rise to a diffuse phlegmon, which, despite the rational treatment, passed to gangrene and was followed by death. The autopsy afforded no evidence either of phlebitis or purulent infection.

In the first of these cases, the phlegmon terminated in resolution; in the second, in suppuration; in the third, in gangrene, and neither during the life of the patient nor after his death were we able to detect any symptom of phlebitis. Thus in nine cases of grave accidents following venesection there were three, or one-third, in which phlebitis was not present. I say *grave* accidents, for in respect to these cases there are numbers of others with which the practitioner daily meets, where a little redness, tumefaction, and even suppuration are found at the place of bleeding, which yield to the most simple means, causing neither the physician nor patient the slightest uneasiness.

2. *The phlebitis which results from venesection is not followed by fatal accidents as often as is believed.* We might cite a large number of cases drawn from our own practice in particular and that of our associates, but we wish to refer only to those observed publicly, and collected, as are all those comprised in this essay, at the clinics of the faculty. These last also number three, but we content ourselves with reporting one which resembles the others *sicut ovum ovo.*

Case IV.—In a man aged 38 years, affected with an intense bronchitis, venesection produced an inflammatory swelling at the bend of the arm. On examination of the part an indurated cord was discovered, painful on pressure, rose colored superficially, knotty, occupying the median cephalic, and extending about five centimeters from the incision towards the cephalic which participated in the inflammation. The mouth of the wound is gaping and grayish; pressure made from above downwards on the venous cord gives issue to a puriform liquid. Repeated applications of leeches made above the disease, according to the directions of Lisfranc, emollient cataplasms, sedatives, and afterwards mercurial unctions, produced in the course of ten or fifteen days the resolution of this localised and probably adhesive phlebitis.

Thus, of six cases of phlebitis, three terminated by resolution. I have myself been attacked with a phlebitis resembling the preceding, being much more mild however, although it gave me great uneasiness, for the fate of the unfortunate Maréchal, whom we knew, who perished a victim to the disease that he was amongst the first so well to describe, was in my memory.

As to recovery from phlebitis supervening at the period of purulent infection, it is fortunate that all practitioners do not encounter it, and I confess, for my own part, that I have never either obtained or observed it. I call this result fortunate, for I am convinced that nature has more to do in these very rare recoveries than our remedies, whatever they may be.

We omit the report of the three cases where traumatic phlebitis passed through all its accustomed phases from the period of purulent infection until death; they offer nothing of particular interest, and it is not with fatal phlebitis that we are occupied. But to speak of the phlebitis occasioned by phlebotomy, I should mention two other cases where purulent infection resulted, in one, from the operation of laryngotomy, and the other, (a remarkable and important circumstance) from simple scarification in a case of anasarca.

But I leave the history of these traumatic accidents that possess nothing new to the reader—except the bearing of some facts, too few certainly, but which may lead to the rudiment of an opinion or presumption in regard to the relative frequency of the various accidents that sometimes follow venesection, and the terminations of the phlebitis that may arise from this operation,—for a subject more novel if not more important; that is, the history of the phlebitis called spontaneous, in opposition to that which results from wounds.

II. *Spontaneous phlebitis, and its mildness attributed to the absence of the contact of the air.* The term *spontaneous* applied to any malady whatever, is a conventional term, expressing that the cause of the disease is unknown; for every morbid phenomenon must have a cause consisting in a modification of the elements of the organism.

During some years past numerous cases of spontaneous phlebitis have occurred among our patients. In none of these, that we now remember, in none, we repeat, have suppuration or accidents from absorption manifested themselves. We have asked ourselves *if the absence of the contact of air* was not the cause of this benignity of the spontaneous and subcutaneous phlebitis unattended by division of the skin. We have always, as now, believed ourselves authorised to consider this kind of affection as mild, compared to that where the inflamed vein is uncovered. This is what I published in 1842, in the summary of the Medical Clinique of the Faculty of Strasbourg. These remarks thrown in a report were passed unnoticed, as do the majority of things produced in the Provinces, and it was with great satisfaction that, three years after, I recognized in a memoir of Dr. Bouchat, published in the Gazette Médicale, of Paris, (April, 1845,) several of these ideas, developed and conveyed in analogous terms. From the fifty-one cases collected, obtained from authors and observed by himself, our *confrere* concludes that spontaneous phlebitis, which he prefers designating as "*phlegmasia alba dolens non-puerperale*," never terminates by suppuration; that it is never followed by accidents of absorption; that of itself it is not a grave affection, and does not of necessity require energetic treatment.

M. Bouchat has wished to ascertain the cause of those phenomena which, according to him, separate this affection from traumatic phlebitis, "and which should prevent the slightest connection being established between them." Upon the latter point we cannot agree with him; first, because we cannot see the essential differences that separate the two affections; second, because it appears in some cases, rare, we will admit, that spontaneous phlebitis does terminate in suppuration.

To return to the formal cause, the author admits that in spontaneous phlebitis complicating chronic affections, which is that most frequently seen, the coagulation of the blood is the primitive fact; that the cause of this coagulation of the blood resides in the relative excess of the fibrine of this fluid; finally, that this phlebitis is produced only secondarily to this coagulation. These opinions have been adopted by the learned author of the *Compendium de Médicine*, article *Phlebite.* For ourselves, we can only see in these propositions hypotheses which do not appear sufficient to sustain the discussion. How, in fact, can we conceive of this spontaneous coagulation of the blood, especially when it occurs in cases where the plasticity of the blood is, according to these authors, diminished, as in typhoid fever? Why does the blood coagulate in certain vessels sooner than in others? If the plasticity of the blood is the real cause of this coagulation, why does it not rather show itself in the acute phlegmasia—in rheumatism, for example, where the fibrin of the blood is, as they again declare, at its maximum quantity? How is it possible to explain this coagulation thus localized, if it is not by a local cause itself? But is it asked, what is the cause of this phlebitis? It is, we reply, that which produces so many phlegmasias, whose cause is unknown to us, and which shows itself so frequently in valetudinarians; such are erysipelas, stomatitis, enteritis, and the pneumonias called ultimate. How demonstrate that phlebitis is consecutive to the coagulation of the blood, when the pain of the limb is, according to the testimony of all observers, the first phenomenon observed? Why credit an exception so opposed to the general rule, that the coagulation of the blood may be the consequence of an alteration of the vessel? Even admitting that phlebitis may be consecutive to the coagulation, is it the less a phlebitis, and does an inflammation change its nature from being secondary?

Such are the reflections that occurred to me while reading the essay of our *confrère*, and I was not convinced either by this absence of the clot in the first periods of the disease, or by the absence of redness of the venous parietes, already signalized by Cruveilhier, who has done most for the history of phlebitis—an absence of redness which is explained in the majority of cases by the period at which the affected part is most frequently observed, that is to say, after the resolution of the phlegmasia, when the obliteration of the vessel is the only remaining vestige of the phlebitis. I persist in considering the thickening, the hardening of the walls of the veins, coincident with the pain and other acute phenomena, as the inflammatory characters; and instead of having recourse to the sanguine diathesis so problematical in its effects if not in its essence, I believe it my duty to adhere to the etiology that I announced in 1842.

Agreeably to my opinion then expressed, the termination by adhesion and the slight danger of spontaneous phlebitis do not depend upon its being secondary, which, I repeat, implies nothing as to the gravity of the disease; these effects appear to me to arise much more probably from the fact that this primitive phlebitis is protected from the air. I am supported in this, firstly, from the simple observation of facts, which teaches us that concealed phlebitis is mild, while open phlebitis is grave in its character; secondly, from every day's experience, which demonstrates the unpleasant effects of the contact of the air with inflamed, and especially suppurating surfaces; thirdly, from those other observations, borrowed from modern surgery, which so clearly establish the advantages of light dressings and immediate reunions; fourthly, and especially, the remarkable results obtained from the surgical practice called subcutaneous. Although we believe we have on our side both direct observation and every analogy, to M. Bouchat belongs the merit of having performed a remarkable labor, and of having lucidly established the coincidence of adhesive phlebitis with chronic affections, which is the principal object of his memoir. I should have kept all these considerations to myself, if a most interesting case, which recently presented itself at my clinic, had not afforded me the opportunity of expressing the opinions which I entertain on this subject, at the same time that I gave that publicity to the case which it deserved.

Among the great number of chronic affections of every variety that have occurred at the clinic of the faculty of Strasbourg during the last eleven years, I have observed only four times in a very manifest manner the complication of spontaneous phlebitis. Of these four examples. there is one of which I have a very imperfect recollection, and of which I cannot find the report among so many others. Another of these facts relates to a phthisical patient, who had varices of the legs, which inflamed during the march of the disease, then passed to resolution, which prevented our wish to establish at the autopsy the state of the previously inflamed veins. We proceed to report the other two cases *in extenso*, which we the more readily undertake, as they are the first and last that we have witnessed:

*Spontaneous Phlebitis, Terminating in Resolution, in a Phthisical Woman; Death; Necroscopy.*

Case V.—A semstress, aged 35 years, entered the clinic on the 18th of August, 1837, having been sick five months. For three months her menses have not appeared. At her entry she had a cough, flocculent expectoration, febrile pulse, dullness, mucous *rales*, cavernous *souffle*, pectoriloquy at the summit of the right lung, night sweats; besides which, she complains of very acute pain in the calf of the left leg, which presents nothing worthy of note except sensibility to pressure.—Gum tisane, decoction of wild cherry-tree bark; ten leeches and emollient cataplasms to the calf of the leg.

For some days following the patient was affected with indigestion from which a diarrhœa resulted. Twelve leeches to the anus, emollients, diet. The fever presented quotidian exacerbations, to arrest which, sulphate of quinine was administered; but the fever persisting and the pulmonary symptoms becoming aggravated, the quinine was suspended. Sixteen leeches under the clavicles, emollients.

The 2d of September, fifteen days after her admission, the pain in the calf continues uninterruptedly; upon the course of the external saphena an unequal and painful cord is discovered; the lower part of the leg is occupied by a slight and resisting engorgement. We recognise phlebitis. Fifteen leeches to the calf, emollient cataplasms.

The following days the pain extended to the thigh. Twelve leeches.

September 8th, the knotty cord of the leg can be felt in the bend of the knee; same pectoral symptoms, continued fever. Fifteen leeches to the popliteal region, blister to the arm, emollients, opium.

Towards the end of the month, the venous cord still remains, notwithstanding repeated local bleedings, but it is less painful; the œdema of the leg is always slight. Early in October the thoracic symptoms grew worse, dyspnœa increasing, frequent cough, emaciation, hectic fever. Small bleeding at the arm, emollients, opium, diet.

The chest absorbs the attention; we no longer occupied ourselves with the leg, in which the patient complains of no more pain. The phthisis made rapid progress, and the patient died on the 26th of October, a little more than two months after her admission into the hospital.

At the autopsy, the lungs were found hollow, with membranous caverns. In the other organs there was no particular lesion; no vestige of purulent absorption.

*Examination of the veins of the left inferior extremity.*—Very light œdema of the leg. The femoral vein presented a perceptible thickening of its parietes; it is obliterated by a consistent and ancient fibrinous clot, which extends above from the external iliac vein downwards in the popliteal and external saphenas to the base of the calf.

One may judge by the activity of our treatment of this spontaneous phlebitis how serious were the apprehensions we entertained; we were indeed alarmed by the dangers which, according to authors, accompany phlebitis, and we are disposed to attribute the resolution to the treatment which we pursued. The pain in the calf, it will be remarked, which was the first symptom of the disease, is observed in the majority of cases of this kind. The œdema was slight compared with what it ordinarily is. There is no doubt in our mind that this was a primitive phlebitis. The two cases previously mentioned in this memoir occurred subsequent to this. The disease assumed the same character of initial pain; the œdema was also slight. In one of them, I have remarked, the phlebitis seized veins already varicose, a circumstance which should confirm us in the opinion that it was a phlebitis with which we had to do, the sub-inflammatory nature of varices, in a majority of cases, being generally admitted since the labors of Breschet, Briquet, etc., and we should believe that an acute inflammation had become engrafted upon a chronic phlogosis.

Notwithstanding the favorable termination in all three of these cases, although the last were treated with extreme moderation, may dissipate our fears respecting the consequences of this spontaneous phlebitis, still they have not taught us to distinguish it from the traumatic form of that disease. These were the facts which formed the basis of the opinions expressed by us in our report of 1842—opinions which have been alluded to above, and are supported in the essay of M. Bouchat.

Finally, some months since, the following case, remarkable in so many respects, presented itself, which, after stripping it of details foreign to the subject in hand, we proceed to report:

*Acute Articular Rheumatism Relieved by Repeated Sanguine Emissions—Typhoid Fever (Follicular Enteritis) Cured by the Expectant Method—Phlegmasia Alba Dolens (Phlebitis) Terminated in Adhesion—Pelvic Ulceration—Exhaustion—Purulent Infection—Death—Necroscopy.*

Case VI.—In the description which I am about to give, I have deemed it preferable to divide the history into four periods:

1st. *Period of rheumatism.*—A woman, æt. 30 years, of robust constitution and sanguine temperament, entered the clinic on the 23d of April, 1846. She had been laboring for five days under acute articular rheumatism; the constitutional and febrile symptoms were such that we ordered frequent bleedings (coup sur coup.) Six bloodlettings were practised in as many days, besides which, forty-four leeches were applied, and the rheumatism was thus relieved on the sixth day of the treatment and the eleventh of the disease.

2d. *Period of follicular enteritis.*—On the tenth day of the disease, signs of typhoid fever manifested themselves. The next day we observed the face dull and sunken, nares contracted, tongue coated and dry, abdomen a little sensible on pressure, pulse 100. Emollients.

The following days the adynamic symptoms persisted, and diarrhœa supervened, with involuntary discharges; typhoid bronchitis, with rales, dyspnœa, etc., now came on.—Sedative emollients.—Excoriations, which insensibly increased, were seen on the sacrum.

On the twenty-fifth day (fifteenth of the typhoid state), the patient complained of pain in the right leg, which was perceptibly œdematous, resisting, and sensible on pressure; pulse 120. Emollient cataplasms upon the right iliac region, tepid acidulated lotions to the leg.

The typhoid condition gradually amended under the influence of the sedative emollients to such a degree, that on the sixteenth day of its duration and twenty-sixth of the disease, we were able to pronounce the patient convalescent from this second disorder.

3d. *Period of phlegmasia alba dolens (phlebitis.)*—The twenty-seventh day, the painful œdema, dating now three days, appeared to have diminished in the leg and concentrated itself at the thigh. The expression of the face was good, the diarrhœa occurred only at intervals, but the pulse remained frequent, and the emaciation progressed.

On the thirtieth day, the superficial veins were defined on the thigh and hypogastrium, and we recognised a deep-seated phlebitis, with obliteration. The excoriations still remained comparatively superficial. Emollients; dress the wound with tannate of lead; soups.

On the thirty-second day, the œdema of the thigh was very decided, the articulation of the knee fluctuating. *Ut supra;* mercurial unctions upon the œdema four times a day.

The thirty-sixth day of the disease, the left leg became infiltrated, twelve days after the appearance of the œdema in the right. The general exhaustion progressed. Wine and water, fomentations of quinine.

The forty-first day the two legs were very œdematous, resisting and shining. The veins of the thigh and abdomen, even as high up as the thorax, form a very distinct network. The diarrhœa continued and the ulceration extended. Rice tisane, abdominal cataplasms, opium, soups, dressing with opiate cerate.

On the following days the œdema diminished, was no longer painful, and on the fiftieth day a little thickening of the legs was all that remained. The superficial venous network was very evident. The *phlegmasia alba dolens* or phlebitis, which continued during twenty-five days, might be looked upon as resolved. The diarrhœa and pelvic ulcerations still persisted. Rice tisane, syrup of quinine, injections with extract of rhatania three scruples and ten drops of laudanum.

4th. *Period of exhaustion and purulent infection.*—From this time the exhaustion, caused by the diarrhœa and pelvic ulcerations, which multiply in spite of the various methods and applications that have been used, steadily progresses. Towards the ninetieth day a new œdema arose, now engendered by the cachexia; the ulcerations penetrated to the bone; the face has become hippocratic; the pulse threadlike. Briefly, notwithstanding the tonics and supporting alimentation the patient, exhausted by the suppuration, diarrhœa, fever, and pain, died on the 28th of July, the hundredth day of the disease, the fiftieth after the resolution of the phlebitis, without my being able precisely to fix the period when the purulent infection, caused by the pelvic ulcers that evacuated themselves at the autopsy, had supervened.

*Necroscopy thirty-six hours after death.*—Extreme marasmus, large and deep ulcers around the pelvis, infiltration of the legs.

*Thoracic cavity.*—Lungs engorged with a sero-sanguinolent fluid. On the external portion of the right lung a dozen metastatic abscesses were observed, varying in volume from the size of a pea to that of a filbert; other abscesses of the same nature were found more deeply seated.

*Abdominal cavity.*—At the end of the small intestine some brown elliptical spots appeared; vestiges of ulcerated or resolved wounds of the ancient follicular enteritis; redness, and fungous condition of the large intestines.

*Examination of the veins of the inferior extremities.*—On both sides the tibial, crural, external, and internal iliac arteries, and even a portion of the primitive iliac, were found obliterated by a long, consistent, fibrinous clot, varying in color from violet, to a bright yellow. These ancient and solid clots adhere slightly to the internal walls of the veins, which are sensibly thickened and offer various discolorations. The internal saphena veins are free up to their insertion into the crural vein, permeable and contain a small quantity of fluid blood. They communicate by large anastomoses with the branches of the epigastric and the tegumentary vessels of the abdomen, which themselves anastomose with the superficial veins of the thorax. *The articulation* presents nothing remarkable.

The following are the principal reflections which this case suggests:

1st. An acute rheumatism has rapidly disappeared under repeated bloodlettings.

2d. To this affection, regarded at present as purely inflammatory, seeing the excess of the fibrin of the blood which characterizes it, succeeds another affection, the inflammatory nature of which is contested, seeing the diminution of the fibrin of the blood so frequently observed in it; that is, typhoid fever.

3d. During the course of this affection, with deficiency of fibrin, the coagulation of the blood in the inferior extremities manifests itself. Is it not more natural to attribute this coagulation to a phlebitis than to plasticity of the blood which we are unable to prove?

4th. Phlebitis resolved itself after having offered all the characters of phlegmasia alba dolens.

5th. Although there existed around the pelvis ulcers that continually increased, it was only a long time after the resolution of the phlebitis (fifty days) that the patient succumbed to exhaustion and purulent infection, which, even posteriorly to the phlebitis of the members, had manifested its source in the pelvic ulcers.

We proceed to detail another observation, taken from the Bulletin de Thérapeutique, (Oct. 1846,) which, while it confirms some of the preceding considerations, furnishes others that are new.

*Phlegmasia Alba Dolens (Phlebitis) occurring in the course of a Typhoid Fever.*

Case VII.—A girl æt. 20 years, entered the Necker Hospital in the service of M. Trousseau, towards the end of August, 1846. She had been already laboring under a well characterized typhoid fever, which was accompanied by certain ataxic phenomena. Saline purgatives were administered, at first, every day, and after a little time at greater intervals, under the influence of which the typhoid symptoms very notably amended. Convalescence seemed upon the point of being established towards the third week, when, one morning at the visit, the patient complained of an extremely sharp pain, which had arisen suddenly during the night, in the left leg. On examination of this part a general serous infiltration of the foot and leg was observed, with tension and resistance of the skin, whose shining color contrasted strongly with that of the limb of the opposite side. The pain was inconsiderable on the anterior portion of the leg, but at the middle of the calf it acquired, especially under pressure, very great intensity. On placing the hand in the popliteal hollow a hard and painful cord could be felt, evidently formed by the obliterated popliteal vein. The limb was engorged throughout, and walking was impossible.

Notwithstanding this complication, convalescence was soon established; the œdema gradually diminished, and although there still remained some traces of it on the 3d of October the patient was nevertheless able to be up the greater part of the day.

Here, again, is a *coagulation* of the blood in a patient with *defibrination.* The author agrees with us in believing it was a primitive phlebitis, pure and simple; and we do think that it cannot be considered as anything else. Another important consideration arising from this and the preceding cases is, that spontaneous phlebitis is not always connected with chronic affections; for in the case in our hands the phlebitis was produced on the twenty-fifth day, and in this at the end of the third week.

It is still further to be remarked, that the subjects of these two cases were in the way of recovery when the phlebitis disclosed itself—that our patient would probably have recovered but for the fatal complication of pelvic ulcers, which supervened fifty days after the resolution of the phlebitis, and that the last patient was positively cured.

Phlebitis, then, is a pure accident, which should not be thought necessarily mortal, and which ought not to be compared, as sometimes it has been, to those "*ultimate*" accidents which announce a speedy dissolution. From these facts, not multiplied it is true, but which are found corroborated by analogous cases abounding in the archives of the science, we believe ourselves authorized to deduce the following conclusions:

1st. Spontaneous phlebitis most generally, but not always, occurs as a complication of chronic affections.

2d. Spontaneous phlebitis is very probably primitive, viz: anterior to the coagulation of the blood.

3d. Spontaneous phlebitis is probably of the same nature as traumatic phlebitis.

4th. If spontaneous phlebitis is almost always adhesive and rarely terminates by suppuration and purulent absorption, it is, very probably, because the inflamed vein is preserved from the contact of the air.

5th. This agency being admitted, very important practical consequences result, for it demonstrates the advantages of immediate reunions in large operations, the necessity of closing exactly the opening of the vein in venesection, the utility of light dressings, and the excellence of the surgical method called subcutaneous.

6th. Spontaneous phlebitis presents the same symptoms, and is very probably of the same nature, as the affection known under the name of *phlegmasia alba dolens;* and that the latter, therefore, is not a disease peculiar to puerperal women, but may arise in the male sex, and under very varied circumstances.

7th. Spontaneous phlebitis, although sometimes giving rise to extensive œdema, is relieved, almost always, and with much ease, by the application of mild remedies.

8th and finally. Spontaneous phlebitis is a simple accident of disease where it occurs as a complication, and does not infallibly warrant an unfavorable prognosis.

The inhalation of ether continues to be the most exciting subject before the professional mind of Paris. At a meeting of the *Cercle Agricole*, to which I procured a ticket through our good friend, M. Ed. de Verneuil, I heard an interesting detail, this evening, of its effects upon the lower animals by M. Constantin James, a young man of fine abilities. I have witnessed operations by Roux, Blandin, and Velpeau, at the Hôtel Dieu and La Charité Hospitals, on patients under the influence of the vapor. Among others, I saw Blandin exsect with the most perfect success two mammary tumors from women narcotised by the ether, neither of whom cried or made any resistance during the operation, nor remembered it after it was over. The same surgeon has employed the ether with like happy effects in a number of other cases; but it is only fair to state, that in some instances, while the subjects appeared to be fully under the influence of the narcotic, they nevertheless felt and complained loudly of the pain. Roux has operated very many times on patients who had been prepared by the ether, and in the majority of cases with the most satisfactory results. With Velpeau the article has proved equally successful, and, in addition to several painful operations performed upon individuals under its influence, he reduced, a few days since, a luxation of the shoulder joint in a man which previously to the inhalation had resisted all efforts at reduction. These are only a few of the cases that have occurred at the hospitals mentioned. Every morning, one may witness similar triumphs of this great discovery in the surgical ward of any of the Paris hospitals. I call it a *great* discovery, and so it is esteemed by the profession here. It is even ranked by some with that which has rendered the name of Jenner immortal. The credit of it generally is conceded to our country, but a counter claim has been set up, and a Frenchman is endeavoring to show, that he was the first to prove that the vapors of ether are narcotic.

The work of Desmarres on the eye is not yet out and will hardly be issued before the beginning of April. I am glad to see that a translation of it by Dr. Clendinen, of Baltimore, is to be published in America contemporaneously with the appearance of the original in Paris. I bespeak for the work the favorable reception of my countrymen, assured by my knowledge of the ability of Dr. Desmarres, that it will be one of the very highest excellence.

Paris, February 27th, 1847.

